# Transport of Acrosomal Enzymes by KIFC1 via the Acroframosomal Cytoskeleton during Spermatogenesis in *Macrobrachium rosenbergii* (Crustacea, Decapoda, *Malacostracea*)

**DOI:** 10.3390/ani12080991

**Published:** 2022-04-12

**Authors:** Le Chang, Qiu-Meng Xiang, Jun-Quan Zhu, Yin-Er Chen, Dao-Jun Tang, Chun-Dan Zhang, Cong-Cong Hou

**Affiliations:** Key Laboratory of Aquacultural Biotechnology Ministry of Education, School of Marine Sciences, Ningbo University, Ningbo 315822, China; 2011091052@nbu.edu.cn (L.C.); 1911091064@nbu.edu.cn (Q.-M.X.); zhujunquan@nbu.edu.cn (J.-Q.Z.); chenyiner@nbu.edu.cn (Y.-E.C.); tangdaojun@nbu.edu.cn (D.-J.T.); zhangchundan@nbu.edu.cn (C.-D.Z.)

**Keywords:** KIFC1, Acrosin, spermatogenesis, *Macrobrachium rosenbergii*, acroframosome

## Abstract

**Simple Summary:**

In crustaceans, the sperm have no tail, and spermatogenesis consists only of acrosomal formation and nuclear deformation. The mechanism of acrosome formation during spermatogenesis of *Macrobrachium rosenbergii* is one of the hot topics in reproductive biology. Many motor proteins are involved in spermatogenesis. KIFC1, as a member of the kinesin family, is one of the motor proteins that our lab has been focusing on. The acrosome contains a large number of acrosomal enzymes for the hydrolysis of the egg envelope. In order to understand how these acrosomal enzymes are transported to the acrosome cap after synthesis, we cloned the KIFC1 and the Acrosin of *M. rosenbergii*. By detecting the localization of KIFC1 and Acrosin, we found that Mr-KIFC1 may be involved in acrosomal enzyme transport during spermiogenesis of *M. rosenbergii*. This study is to propose the function of KIFC1 to transport acrosomal enzymes along the acroframosome structure during crustacean spermatogenesis.

**Abstract:**

The spermatogenesis of crustaceans includes nuclear deformation and acrosome formation. The mechanism of acrosome formation is one focus of reproductive biology. In this study, *Macrobrachium rosenbergii* was selected as the research object to explore the mechanism of acrosome formation. The acrosome contains a large number of acrosomal enzymes for the hydrolysis of the egg envelope. How these acrosomal enzymes are transported to the acrosomal site after synthesis is the key scientific question of this study. The acroframosome (AFS) structure of caridean sperm has been reported. We hypothesized that acrosomal enzymes may be transported along the AFS framework to the acrosome by motor proteins. To study this hypothesis, we obtained the full-length cDNA sequences of *Mr-kifc1* and *Mr-Acrosin* from the testis of *M. rosenbergii*. The *Mr-kifc1* and *Mr-Acrosin* mRNA expression levels were highest in testis. We detected the distribution of Mr-KIFC1 and its colocalization with Mr-Acrosin during spermatogenesis by immunofluorescence. The colocalization of Mr-KIFC1 and microtubule indicated that Mr-KIFC1 may participate in sperm acrosome formation and nucleus maturation. The colocalization of Mr-KIFC1 and Mr-Acrosin indicated that Mr-KIFC1 may be involved in Acrosin transport during spermiogenesis of *M. rosenbergii*. These results suggest that Mr-KIFC1 may be involved in acrosomal enzymes transport during spermiogenesis of *M. rosenbergii*.

## 1. Introduction

Spermatogenesis is a highly ordered and complex physiological process that is coordinated by a variety of cytokines and signaling pathways and can be divided into three stages: spermatogonial mitosis, spermatocyte meiosis, and spermiogenesis [1,2]. This process involves a series of important cellular biological changes that are crucial to an animal’s ability to reproduce. Spermatogenesis is a process in which haploid sperm cells form fertilized sperm through a series of complex differentiation and morphological changes, including nuclear remodeling, acrosome formation, and tail formation [3]. However, this does not mean that all organisms will undergo such changes. The mature sperm of most decapods have a unique morphology, showing irregular shape, no flagellum, and no swimming state [4,5,6]. However, there are also great differences in sperm morphology among Decapoda. For example, mature sperm of *E. sinensis* and *P. trituberculatus* in suborder Reptantia are composed of acrosomes, nuclei, and radiative arms, and the nucleus is cup-shaped and wrapped around the apical periphery [5,7]. In the swimming suborder, the acrosome of mature sperm of *M. nipponense* is an evaginated umbrella-shaped structure enclosing a discoid nucleus [8], and the acrosome of mature spermatozoa is funnel-shaped and upside-down in the nucleus [9].

Fertilization in animals is a highly ordered process, including the specific binding of the sperm to the egg, the induction of the acrosome reaction, and gamete fusion [10]. Flagellated sperm are attracted by secretions from eggs of the same species. However, the mature sperm of crustaceans do not have flagella, and the process of sperm and egg recognition is complex and passive, which is quite different from the fertilization mode of mammals. This process and changes in sperm morphology have been described in a number of articles [11,12]. In decapods within Reptantia, fertilization depends on acrosomal tubule attraction on the sperm surface [5], while fertilization in swimming decapods depends on the acrosome and AFS [11,13,14]. At the middle stage of spermiogenesis, the endoplasmic reticulum, mitochondria, and Golgi vesicles gather together to form a temporary organelle structure, the lamellar complex (LCx). At the same time, driven by centrosomes, a special cytoskeletal structure is gradually formed, which is called the AFS. The AFS is a structure unique to the sperm of the true shrimp suborder and provides a track for motor proteins to transport cargoes to participate in acrosomal accumulation and acrosome formation [15]. Mature sperm of *M. rosenbergii* consist of two parts: a dense anterior part and a spherical posterior part. The front part is the acrosome with a valgus umbrella-like structure. The posterior portion is a transparent globular structure composed of cytoplasm and crescent-shaped nuclei [16]. Studies have suggested that there are various hydrolases on the spiker that function as acrosomes and assist sperm in penetrating the ovum [13,17]. This study aimed to investigate the AFS structure that mediates Acrosin transportation during spermatogenesis to facilitate acrosome formation and promote sperm-egg fusion.

There are many hydrolases in the acrosome. Acrosin is a hydrolase that exists in the form of inactive proacrosomal precursors in organisms and is activated by specific proteolysis during acrosome reactions [18]. Acrosin, as a trypsin-like serine protease unique to the sperm acrosome, plays an important role in the acrosome reaction and the binding and penetration of the zona pellucida between sperm and ovum [19]. In addition, it assists the release of other enzymes in the acrosome. It can enhance sperm activity and promote sperm motility [20,21]. Therefore, Acrosin directly affects sperm motility and fertilization [22,23,24]. However, how Acrosin is transported to acrosomal sites after synthesis is still unknown.

Kinesins are motor proteins that use microtubules as a track and transport cargoes. It releases energy by hydrolyzing ATP (adenosine triphosphate) to efficiently and accurately transport the carried cargoes (including membrane organelles, protein complexes, vesicles, RNA, and dsDNA) to specific parts of the cell to perform various functions [25,26,27]. Kinesins are present in a wide variety of organisms and participate in a large number of biological activities, such as flagellum swimming, cilia beating, and spindle and chromosome movement during neuronal development, mitosis, and meiosis [28,29,30]. To date, 14 kinesin family proteins and 1 ungrouped motor protein have been characterized [31]. KIFC1 is a member of the kinesin-14 family and has three functional domains, including heads with ATP and microtubule binding sites composed of two spherical structures, a rod-shaped stem region composed of heavy chains, and a fan-shaped tail composed of heavy chains and light chains used to transport different goods (fan-like end). It has been proven that it can participate in acrosome formation in different organisms, including *Rattus norvegicus* [32], *Eumeces chinensis* [33], *Octopus tankahkeei* [34], *Larimichthys crocea* [35], *Portunus trituberculatus* [36], *Exopalaemon modestus* [14], *Penaeus (Marsupenaeus) japonicas* [37], and *Phascolosoma esculenta* [38]. Based on this previous research, this paper hypothesized that kinesin KIFC1 participates in acrosome formation and nuclear shaping during the formation of *M. rosenbergii* sperm and can transport different proteases to the designated position of the acrosome along the AFS framework to promote the formation of the acrosome. To test our hypothesis, at the gene level, we cloned the full-length cDNA of *Mr-kifc1* and *Mr-Acrosin*, analyzed their domains, and studied the expression distribution of the two genes during spermatogenesis. At the protein level, we studied the expression and distribution of Mr-KIFC1 and hydrolase during spermatogenesis by immunofluorescence. This study provides evidence for further studies on the mechanism of spermatogenesis in decapods.

## 2. Materials and Methods

### 2.1. Preparation of Animals and Samples

The *M. rosenbergii* used in our experiments were purchased from the Meixi Village aquatic market (Ningbo, China). We sampled 20 male *M. rosenbergii* shrimps each time (once every month) from May to July 2021. Mature *M. rosenbergii* shrimps were selected for our experiments. Shrimps were dissected on ice, and testicular, hepatopancreas, heart, gill, and muscle tissues were detached, quickly frozen in liquid nitrogen, and then stored at −80 °C for total RNA and protein extraction. A part of fresh testis was placed in 4% PFA-PBS (pH 7.4) and fixed at 4 °C for 3–4 h. After that, 1 × PBS was used to wash off the fixation solution on the tissue surface (repeated 3 times), and 0.5 mol/L sucrose solution was added to infiltrate the tissue (overnight treatment at 4 °C) in an RNA-free atmosphere. The fixed tissues were then embedded in the O.C.T. complex (SAKURA, Torrance, CA, USA), frozen at −20 °C, and finally cryopreserved at −80 °C for subsequent immunofluorescence and in situ hybridization experiments.

No special permission is required for the use of *M. rosenbergii* as a laboratory animal in China.

### 2.2. Full-Length cDNA Cloning of Mr-kifc1 and Mr-Acrosin

Total RNA was extracted from different tissues of *M. rosenbergii* using TRIzol reagent (Tiangen Biotech, Beijing, China), and the obtained RNA was used for normal reverse transcription using the PrimeScript^®^ RT Reagent Kit (Takara, Dalia, China). A SMARTer RACE 5′/3′ Kit (Takara, Dalian, China) was used to synthesize 3′ and 5′ terminal cDNA from the testis of *M. rosenbergii*. All the above operations were carried out according to the instructions provided by the manufacturer.

The kifc1 cDNA sequences of *Homo sapiens* (NM_002263.3), *Mus musculus* (NM_001195298.1), *Xenopus laevis* (NM_001087534.1), *Danio rerio* (NM_001044954.2), *M. nipponense* (JN645278.1), *Palaemon modestus* (KF728597.1), *Portunus trituberculatus* (KT161948.1), and *Eriocheir sinensis* (GU990077.1) were downloaded from the National Center for Biotechnology Information (https://www.ncbi.nlm.nih.gov/, accessed on 1 December 2014, NCBI) database, and the conserved sequence was obtained by Vector NTI10 (Invitrogen, CA, USA) alignment. The sequences were imported into Primer Premier 5 software (Premier Biosoft International, Palo Alto, CA, USA) for the design of degenerate primers and sent to Zhejiang Youkang Biotechnology Co., Ltd. (Youkang, China) for synthesis. The testis cDNA of *M. rosenbergii* was used as a template, and the Touchdown PCR (TD-PCR) program was used to amplify the intermediate fragments of *Mr-kifc1*. TD-PCR was conducted as follows: 94 °C for 5 min; 8 cycles of 94 °C for 30 s, 55 °C for 30 s (decreased by 0.5 °C/cycle), and 72 °C for 90 s; 27 cycles of 94 °C for 30 s, 51 °C for 30 s, and 72 °C for 90 s; then 72 °C for 10 min for the final extension. The PCR products were separated and identified by 1.0% gel electrophoresis (0.1% nucleic acid dye) after cutting the expected size of the band, and a Quick-type DNA Gel Extraction Kit (BioTeke, Beijing, China) was used to extract DNA fragments. The purified fragments were cloned into pMD18-T-vectors (Takara), propagated in *Escherichia coli* DH5α, and sequenced by Zhejiang Youkang Biotechnology Co., Ltd. (Youkang, China).

Primer Premier 5 software was used to design the specific primers on the intermediate fragment for 5′ and 3′ RACE. The TD-PCR was conducted as follows: 94 °C for 5 min; 8 cycles of 94 °C for 30 s, 69 °C for 30 s (decreased by 0.5 °C/cycle), and 72 °C for 90 s; 27 cycles of 94 °C for 30 s, 65 °C for 30 s, and 72 °C for 90 s; then 72 °C for 10 min for the final extension. The PCR products were treated and sequenced as described above. Finally, the correct fragment sequences were assembled with reference to the sequence in NCBI in Vector NTI10 (Invitrogen) to obtain the full-length cDNA sequence of *Mr-kifc1*.

The same method as above was used to obtain the full-length cDNA sequence of *Mr-Acrosin*. The TD-PCR for the synthesis of intermediate fragments was conducted as follows: 94 °C for 5 min; 8 cycles of 94 °C for 30 s, 59 °C for 30 s (decreased by 0.5 °C/cycle), and 72 °C for 60 s; 27 cycles of 94 °C for 30 s, 55 °C for 30 s, and 72 °C for 60 s; then 72 °C for 10 min for the final extension. The TD-PCR for the synthesis of 5′ and 3′ fragments was conducted as follows: 94 °C for 5 min; 8 cycles of 94 °C for 30 s, 69 °C for 30 s (decreased by 0.5 °C/cycle), and 72 °C for 90 s; 27 cycles of 94 °C for 30 s, 65 °C for 30 s, and 72 °C for 90 s; and then 72 °C for 10 min for the final extension. All the primers used in the study are presented in Table 1.

### 2.3. Multiple Sequence Alignment, Phylogenetic Evolutionary Tree Analysis and Protein Structure Prediction

The putative protein sequences of Mr-KIFC1 and Mr-Acrosin were obtained by the online sequence processing toolkit SMS (http://www.bio-soft.net/sms/, accessed on 10 March 2021). The isoelectric point and molecular weight of Mr-KIFC1 and Mr-Acrosin were predicted by the ExPASy-ProtParam tool (https://web.expasy.org/protparam/, accessed on 12 March 2021). Multiple homologous protein sequences of Mr-KIFC1 were downloaded from the NCBI website, and Vector NTI10 software was used to compare homology. The coiled-coil and motor domains of Mr-KIFC1 and Mr-Acrosin were analyzed using SMART (http://smart.embl-heidelberg.de/, accessed on 18 March 2021). At the same time, the ATP binding sites and microtubule binding sites of Mr-KIFC1 were analyzed by the Conserved Domains Database (CDD) Tools (https://www.ncbi.nlm.nih.gov/cdd, accessed on 24 March 2021) in NCBI. The phylogenetic relationships among the kifc1 sequences were determined using the neighbor-joining (NJ) method and the MEGA 7 program. In addition, the presumed tertiary structure was established for KIFC1 using the I-TASSER online website (https://zhanglab.ccmb.med.umich.edu/I-TASSER/, accessed on 28 March 2021).

### 2.4. Semiquantitative PCR Analysis of Mr-kifc1 and Mr-Acrosin mRNA Expression

A pair of primers (KIFC1-BDLF, KIFC1-BDLR) were designed by Primer Premier 5 software to analyze the expression of *Mr-kifc1* in the testis, hepatopancreas, heart, gill, muscle, and intestines, with *β-actin* used as an internal control gene. Additionally, a pair of primers (Acrosin-BDLF, Acrosin-BDLR) were designed by Primer Premier 5 software to analyze the expression of *Mr-Acrosin* in the testis, hepatopancreas, heart, gill, and muscle, with *β-actin* used as an internal control gene. We used four individuals and performed three technical replicates. The PCR program was as follows: 94 °C for 5 min; 32 cycles of 94 °C for 30 s, 56 °C for 30 s, and 72 °C for 30 s; and 72 °C for 10 min.

After gel electrophoresis of the PCR products, the images were photographed using a gel imaging system, and greyscale values were analyzed using ImageJ software. One-way ANOVA was performed on the data using LSD, Tukey HSD, Tukey s-b, and Duncan’s tests in IBM SPSS software, and graphs were made using GraphPad Prism 8.0.2 software.

### 2.5. In Situ Hybridization (ISH)

#### 2.5.1. Riboprobe Synthesis

A pair of primers was used to amplify the target cDNA fragment (569 bp) for riboprobe synthesis. The PCR program was as follows: 94 °C for 5 min; 32 cycles of 94 °C for 30 s, 56 °C for 30 s, and 72 °C for 30 s; and 72 °C for 10 min. PCR products were ligated to the PGEM-T EASY vector (Promega, Beijing, China). Afterwards, the ligation products were transferred into *E. coli* DH5α and positive bacteria were screened by the blue–white spot screening method. Linearization was performed using the corresponding enzyme, followed by purification and use as a template for probes. The fragment was transcribed with a T7 promoter in vitro. After the synthesized RNA was precipitated by ethanol and LiCl, it was resuspended in DEPC-treated H_2_O. Finally, a spectrophotometer and nucleic acid electrophoresis were used to assess the concentration and quality of the riboprobes.

#### 2.5.2. Prehybridization and Hybridization

The frozen sections were removed and dried on a sterile ultraclean table for 10 min, fixed with 4% PFA-PBS solution added dropwise for 10 min, and washed twice with 1 × DEPC-PBS solution for 10 min each time. After that, the sections were equilibrated in 5 × SSC (sodium chloride 0.75 M, sodium citrate 0.075 M, pH 7.0) for 15 min, and then the slides were covered with prehybridization solution (100 μL/slice) and covered with parafilm film. The prehybridization solution was composed of 50% deionized formamide, 40 mg/mL denatured salmon sperm DNA, and 5 × SSC solution. Then, the sections were placed in a wet cassette containing wet cassette solution and prehybridized in a 58 °C water bath for 2 h. Approximately 300 ng/mL denatured and digoxigenin (DIG)-labelled nucleoprotein probe was added to the prehybridization solution to obtain a hybridization buffer. The sections were placed in the hybridization buffer at 57 °C overnight. The sections were then rinsed in 2 × SSC for 30 min at room temperature, followed by 2 × SSC for 1 h at 65 °C and 0.1 × SSC for 1 h at 65 °C.

#### 2.5.3. Detection of the Product Signal

Sections were infiltrated in buffer Ⅰ and washed for 5 min. The slides were incubated with digoxin antibody (DIG buffer Ⅰ dilution) containing 0.5% blocker coupled to alkaline phosphatase at room temperature for 2 h. Stained slides were washed with DIG buffer Ⅰ three times for 15 min each to remove unbound antibodies. Then, the slides were wetted in buffer Ⅱ for 5 min. Color development solution (450 µg/mL nitro blue tetrazolium chloride (NBT) and 170 µg/mL 5-bromo-4-chloro-3-indole-phosphate (BCIP) in buffer Ⅱ) were added dropwise to the slides and incubated at room temperature with protection from light overnight. The color development reaction was terminated by adding TE buffer, and the slides were gently shaken by immersion in 95% ethanol to remove nonspecific staining. The slides were then washed in deionized water for 15 min to remove Tris, dehydrated through a gradient alcohol series (50, 70, 95, 100%) twice at each concentration for 15 min for each step, cleared in xylene, sealed with neutral resin, dried, and then placed on a Nikon Eclipse E80i microscope (Nikon, Tokyo, Japan) for observation and photography.

### 2.6. Antibodies

#### 2.6.1. Prokaryotic Expression

Based on the amino acid sequences of Mr-KIFC1 and Mr-Acrosin, combined with their protein binding properties, we used the antigenic epitope analysis tool (http://www.detaibio.com/tools/epitope-prediction.html, accessed on 10 March 2020) to predict the antigenic fragment. The *Mr-kifc1* fragment was 801 bp (encoding 267 amino acids, approximately 29.77 kDa), and the *Mr-Acrosin* fragment was 492 bp (encoding 164 amino acids, approximately 22.98 kDa). BamHI and EcoRI (Takara, Beijing, China) cut sites were added at the two ends of the *Mr-kifc1* fragment, and EcoRI and SalI (Takara, Beijing, China) cut sites were added at the two ends of the *Mr-Acrosin* fragment. The cloned correct DNA fragment was subjected to a double digestion reaction with plasmid pET28-a (+) (given by the sperm laboratory of Zhejiang University) and then ligated with T4 ligase (Takara, Beijing, China). Positive monoclonal bacteria were screened by liquid culture with shaking, and plasmid extraction was performed with the Plasmid Extraction Mini Kit (Solarbio, Beijing, China). The correct recombinant plasmids pET28-a (+)-HMRK and pET28-a (+)-HLXA were obtained after sequencing, transferred into the Transetta strain (empty pET-28a (+) plasmid was used as a control), and inoculated into 10 mL Kana^+^ liquid medium to expand the culture (37 °C, 220 g). When their OD value reached 0.4–0.6, 1 mM IPTG solution was added to induce recombinant protein expression (37 °C, 220 g, 8 h). The bacterial solution was resuspended after sedimentation, ultrasonically crushed, and centrifuged (10,000 *g*, 4 °C, 15 min). Purification of inclusion body proteins from precipitates was performed using a His-tagged protein purification kit (Beyotime, Shanghai, China) according to the manufacturer’s instructions.

The purified fusion proteins (purity >85%, concentration >1 mg/mL, a total of 5 mg protein) were sent to Hangzhou Hua’an Biotechnology Company (Hangzhou, China) for the preparation of Mr-KIFC1 rabbit polyclonal antibody and Mr-Acrosin rabbit polyclonal antibody. α-Tubulin antibody (anti-mouse) was purchased from Beyotime.

#### 2.6.2. Western Blot Analysis

A 50–100 mg sample of testis was removed, cut into pieces, and then homogenized in 1 mL of cold RAPI (containing 10 μL PMSF) (Beyotime) 3 times in an ice bath with a homogenizer at 5 s intervals each time. The homogenate was then centrifuged at 15,294 *g* for 10 min at 4 °C. The supernatant was carefully transferred to a new precooled centrifuge tube to obtain the total protein extract. The specificity of the Mr-KIFC1 rabbit polyclonal antibody was checked by Western blotting according to the method of Hou and Yang [14], and only one protein band was detected; its molecular weight was 60–75 kDa, consistent with the predicted molecular weight of Mr-KIFC1 (74.55 kDa).

### 2.7. Immunofluorescence (IF)

The testis of *M. rosenbergii* were cut into 6 μm slices on adhesive microscope slides (Citotest, Jiangsu, China) using a frozen sectioning machine and stored at −80 °C. The sections were removed and dried at room temperature for 20 min and permeabilized with 0.3% PBST (made of Triton X-100) for 20 min. A sufficient amount of 5% BSA-PBS was added and covered lightly with sealing film; then, the slides were placed in a humid chamber and incubated at 37 °C for 1.5 h. The blocking solution was discarded, and a sufficient amount of primary antibody (anti-KIFC1 1:500 in PBST/anti-Acrosin 1:500 in PBST and anti-α-Tubulin 1:500) was added. The sections were covered lightly with sealing film and incubated overnight in a humid chamber at 4 °C. Only antibody dilution solution was added to the negative control. The sections were washed 3 times with 0.1% PBST for 10 min each time. After the wash solution was removed, the sections were incubated with Fluor 555-labelled goat anti-rabbit IgG (H + L) (Beyotime, Shanghai, China) and Alexa Fluor 488-labelled donkey anti-mouse IgG (H + L) (Beyotime, Shanghai, China) secondary antibodies for 40–60 min (dilution ratio 1:500), always protected from the light, beginning at this step. The sections were washed 6 times with 0.1% PBST for 10 min, each time avoiding light. The nuclei were stained with a sufficient amount of DAPI (Beyotime, Shanghai, China) and incubated for 5–10 min at room temperature, avoiding light. After rinsing with 0.1% PBST, 10 μL of antifade mounting medium (Beyotime, Shanghai, China) was added, and a microscope cover glass was used to seal the slides (avoiding air bubbles) at 4 °C overnight. Finally, a Zeiss laser scanning confocal microscope (LSM880, Carl Zeiss, Oberkochen, Germany) was used to observe the distribution of Mr-KIFC1 and Mr-Acrosin and the colocalization status of Mr-KIFC1 and AFS.

## 3. Results

### 3.1. The Major Features of Mr-kifc1 and Mr-Acrosin

**Figure 1:**
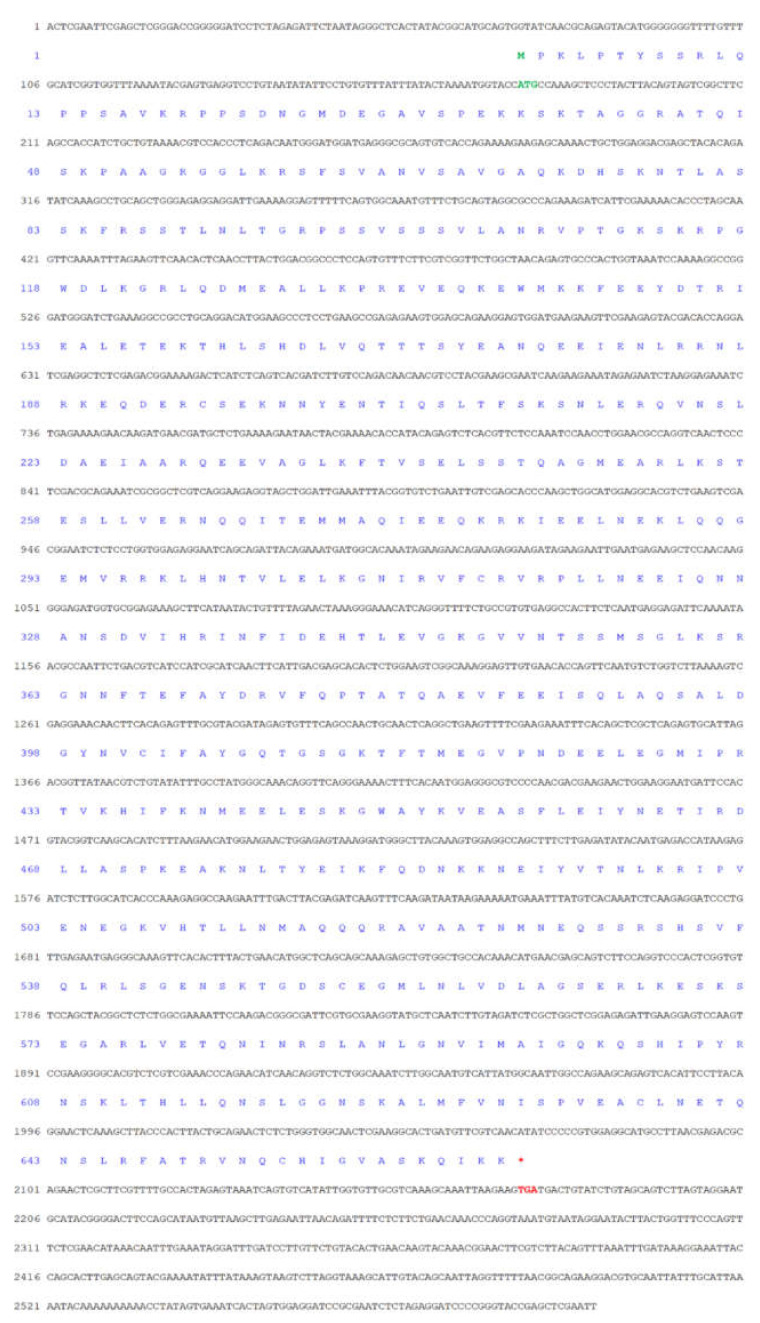
Full-length cDNA and amino acid sequence of *kifc1* in *M. rosenbergii*. The bases highlighted in green represent the start codon, and the bases highlighted in red represent the stop codon. *Mr-kifc1* has a full length of 2603 bp and an open reading frame (ORF) length of 1995 bp, encoding 665 amino acids. The 5’ untranslated region (5’ UTR) is 176 bp, and the 3’ untranslated region (3’ UTR) is 432 bp.

**Figure 2:**
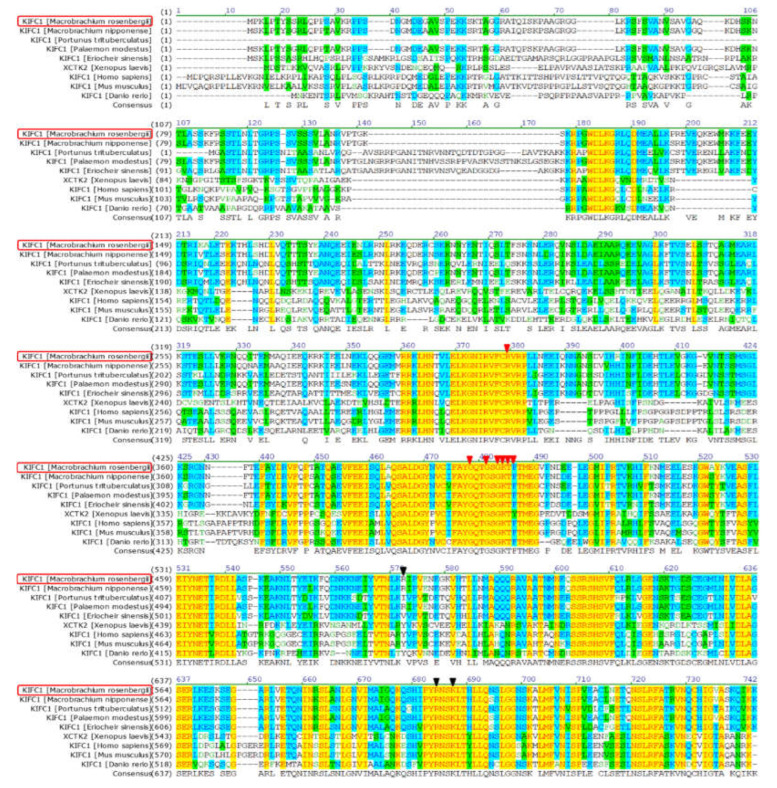
Comparison of the predicted amino acid sequence of *M. rosenbergii* KIFC1 with homologous sequences from other species. ATP binding sites are indicated in red triangles and microtubule binding sites are indicated in black triangles. Mr-KIFC1 shows 93.8, 91.3, 58, 56.8, 39.2, 37.9, 37.9, and 37.3% aa identity with its homologues in *M. nipponense*, *P. modestus*, *E. sinensis*, *P. trituberculatus*, *D. rerio*, *X. laevis*, *H. sapiens,* and *M. musculus*, respectively.

**Figure 3:**
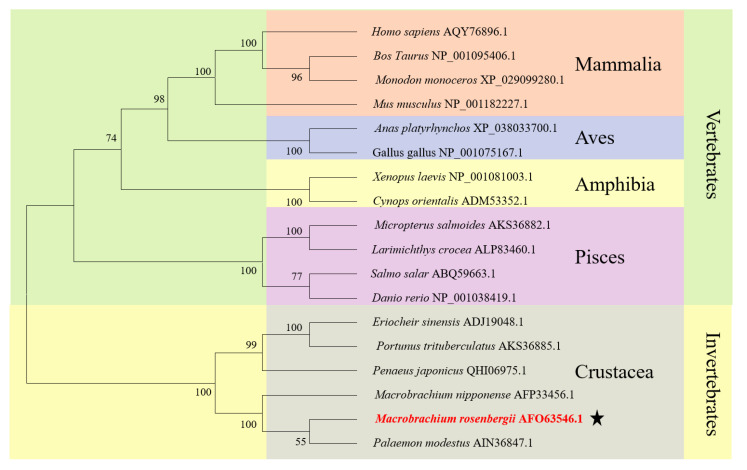
Phylogenetic tree analysis of KIFC1 in *M. rosenbergii*. The phylogenetic tree, which includes species from Mammalia, Aves, Amphibia, Pisces, and Crustacea, was constructed using the neighbor-joining method in MEGA 6. The putative KIFC1 of *M. rosenbergii* constitutes a sister clade with its homologue in *P. modestus*. The star highlighted the evolutionary status of *Mr-kifc1*.

**Figure 4:**
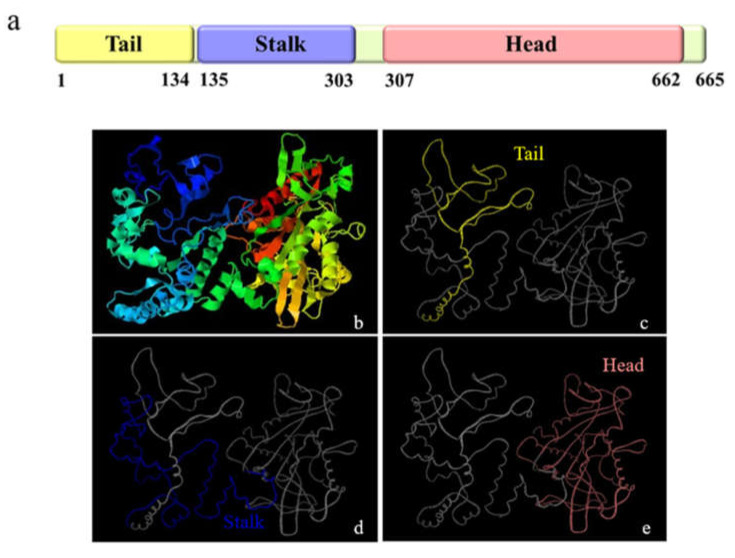
Predicted protein structural analysis of KIFC1 from the testis of *M. rosenbergii.* (**a**) The figure shows the tail (yellow area), stem (blue area), and head (pink area) domains of Mr-KIFC1. The stalk region forms a helix region. (**b**–**e**) The tertiary structure of Mr-KIFC1 predicted by I-TASSER software.

The full-length *Mr-kifc1* (GenBank accession number: JN627516.1) cDNA is 2603 bp, containing a 1995 bp open reading frame (ORF) encoding 665 amino acids (aa), a 176 bp 5′ untranslated region (UTR), and a 432 bp 3′UTR (Figure 1). The molecular weight of Mr-KIFC1 was 74.55 kDa, and its isoelectric point was 8.80. The similarities of the Mr-KIFC1 protein sequence with its homologous sequences in *M. nipponense* (AFP33456.1), *P. modestus* (AIN36847.1), *E. sinensis* (ADJ19048.1), *P. trituberculatus* (AKS36885.1), *D. rerio* (NP_001038419.1), *X. laevis* (NP_001081003.1), *H. sapiens* (AQY76896.1), and *Mus musculus* (NP_001182227.1) were 93.8, 91.3, 58, 56.8, 39.2, 37.9, and 37.9% (Figure 2). The head region of Mr-KIFC1 is more conserved, with ATP binding sites and microtubule binding sites. Phylogenetic tree analysis showed that the predicted Mr-KIFC1 was more closely related to those of invertebrates and more distantly related to those of vertebrates (Figure 3). The secondary structure of Mr-KIFC1 consists of three main structural domains: 1–134 aa for the tail structural domain that binds and transports different cargoes; 135–303 aa for the heavy chain rod-like stem region; and 307–662 aa for the motor structural domain that binds to microtubules (Figure 4).

**Figure 5:**
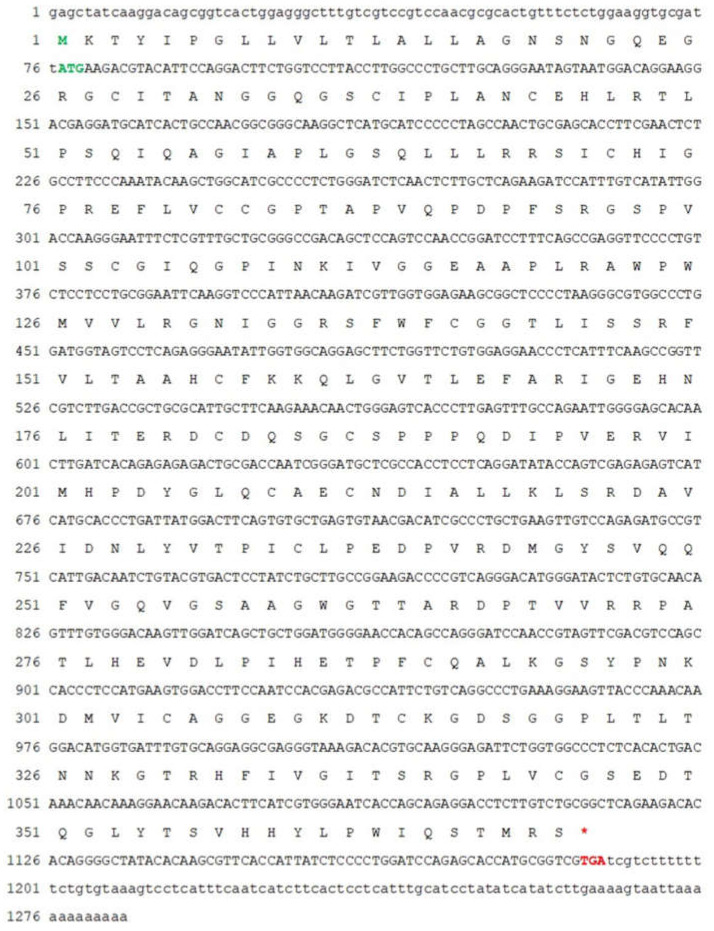
Full-length cDNA and amino acid sequence of *Acrosin* in *M. rosenbergii*. The green and red bold text show the start codon and stop codon, respectively. The full-length *Mr-Acrosin* is formed from a 76 bp 5′ untranslated region (UTR), a 95 bp 3′ untranslated region, and an 1110 bp open reading frame that encodes 370 amino acids.

**Figure 6:**
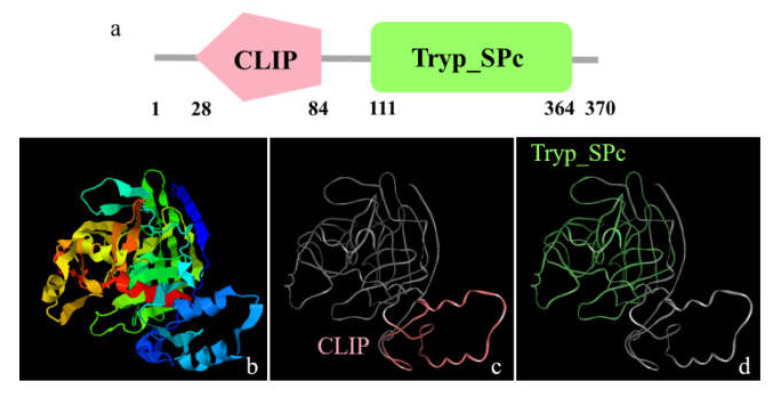
Predicted protein structural analysis of Mr-Acrosin from the testis. (**a**) Two domains of Mr-Acrosin, including the clip domain (pink region) and Tryp-SPc domain (green region). (**b**–**d**) The tertiary structure of Mr-Acrosin predicted by I-TASSER software. CLIP domain contains 57 amino acids (28–84 aa). Tryp-SPc domain covers 254 amino acids (111–364 aa).

The full-length *Mr-Acrosin* (GenBank accession number: OL840042) cDNA is 1573 bp, including an 1110 bp open reading frame (ORF), encoding 370 amino acids with an estimated molecular weight of 40 kDa (Figure 5) and a predicted isoelectric point (pI) of 7.48, 76 bp 5′ UTR, and 95 bp 3′ UTR. The secondary structure of Mr-Acrosin includes the regulatory structural domain clip and the trypsin family serine protease structural domain Tryp-SPc (Figure 6). The structural domain clip is only found in arthropods [39].

### 3.2. Mr-kifc1 and Mr-Acrosin mRNA Expression in Different Tissues of M. rosenbergii

**Figure 7:**
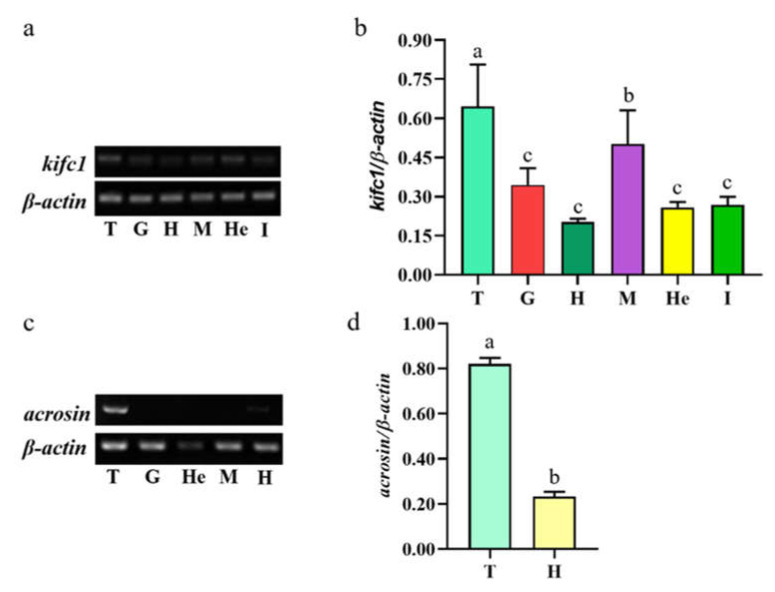
The expression of *Mr-kifc1* and *Mr-Acrosin* in different tissues. (**a**) The above figure shows the expression level of *Mr-kifc1* in different tissues. The lower picture shows *β-actin* as a positive control. (**b**) The relative expression of *Mr-kifc1* mRNA in different tissues was analyzed statistically, and the result was rendered via Image J. The highest expression of *Mr-kifc1* appeared in the testis. (**c**) The above figure shows the expression level of *Mr-acrosin*. The lower picture shows *β-actin* as a positive control. (**d**) The relative expression of *Mr-Acrosin* mRNA in different tissues was analyzed statistically. The highest expression of *Mr-Acrosin* appears in the testis. T: testis, G: gills, H: heart, M: muscle, He: hepatopancreas, I: intestine.

*Mr*-*kifc1* was widely expressed in the testis, muscle, heart, hepatopancreas, gills, and intestine, and its expression was higher in the testis and muscle and lower in the heart (Figure 7a,b). *Mr*-*Acrosin* was only expressed in the testis and heart, and it was highly expressed in the testis (Figure 7c,d).

### 3.3. The Spatial and Temporal Expression Pattern of Mr-kifc1 during Spermatogenesis of M. rosenbergii

**Figure 8:**
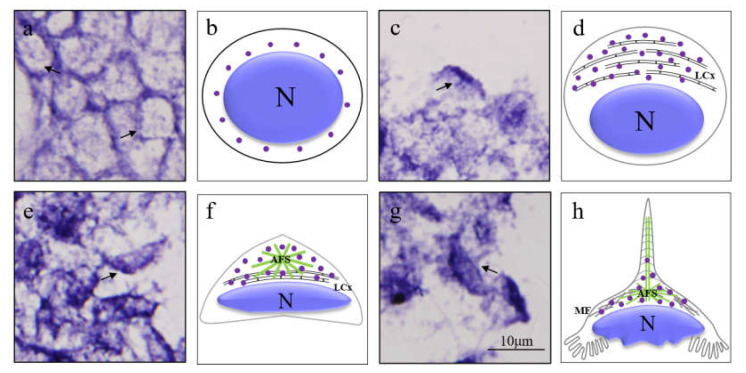
In situ hybridization showing the spatial and temporal expression pattern of *Mr-kifc1* mRNA in *M. rosenbergii* during spermatogenesis. (**a**) Early spermatid: *Mr-kifc1* mRNA signals were distributed in the cytoplasm surrounding the nucleus. (**c**) Middle spermatid: *Mr-kifc1* mRNA signals were obviously concentrated in the cytoplasm at the front of the nucleus. (**e**) Late spermatid: *Mr-kifc1* mRNA signals were concentrated in the AFS structure. (**g**) Mature sperm: *Mr-kifc1* mRNA signals were distributed in the whole AFS structure, but the signals in the peak were weak. (**b**,**d**,**f**,**h**) Diagram. The diagrams show the distribution of *Mr-kifc1* mRNA signals (purple dots) in *M. rosenbergii* during spermatogenesis. MF: microfilaments, N: nucleus (blue), LCx: lamellar complex, AFS: acroframosome (green) and the arrow indicated that the typical signals of *Mr-kifc1*. Scale bar = 10 μm.

We used the anti-sense DIG-labelled *Mr-kifc1* probe to track the spatial and temporal expression pattern of *Mr-kifc1* mRNA in the testis. In early spermatids, *Mr-kifc1* mRNA signals were randomly distributed in the cytoplasm surrounding the nuclei of round sperm cells. At the same time, weak *Mr-kifc1* signals also appeared in the nucleus (Figure 8a,b). In the middle spermatids, the nucleus gradually became ellipsoid and localized to one side of the sperm cell. *Mr-kifc1* mRNA signals were obviously concentrated in the cytoplasm at the front of the nucleus, where the LCx complex was gradually formed (Figure 8c,d). In late spermatids, the AFS structure gradually appeared, and the nucleus became crescent-shaped. *Mr-kifc1* mRNA signals were strongly concentrated in the acrosomal cap of the AFS structure (Figure 8e,f). In mature sperm, the AFS structure gradually extends to a peak. *Mr-kifc1* mRNA signals were distributed in the whole AFS structure, but the signals in the peak were weak (Figure 8g,h). *Mr-kifc1* mRNA was closely related to acrosome formation during spermatogenesis in *M. rosenbergii*.

### 3.4. Validation of the Anti-Mr-KIFC1 Antibody and Anti-Mr-Acrosin Antibody

**Figure 9:**
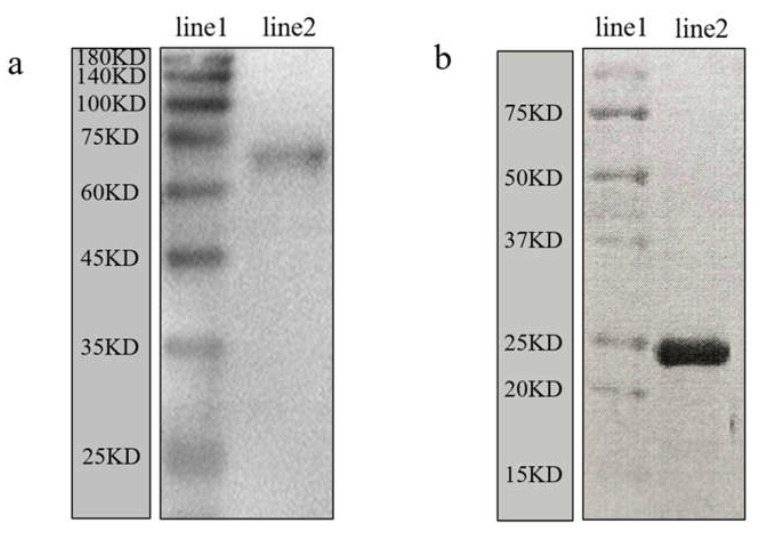
The specificity of the antibody was tested by Western blotting. (**a**) Western blot analysis using the rabbit anti-Mr-KIFC1 antibody. Line 1 shows the protein markers. Line 2 shows that there was only one protein band with a molecular weight of 60–75 kDa, consistent with the predicted molecular weight of Mr-KIFC1 (74.55 kDa), indicating the specificity of the antibody. (**b**) Specificity test of rabbit anti-Mr-Acrosin antibody. Line 1 shows the protein markers. Line 2 shows the purified recombinant protein with a molecular weight of approximately 23 kDa.

The specificity of the rabbit anti-Mr-KIFC1 antibody and anti-Mr-Acrosin antibody was elucidated by Western blotting. The results in Figure 9a indicate that there was only one protein band, which was between 60 and 75 kDa, consistent with the predicted molecular weight of Mr-KIFC1 (74.55 kDa), confirming the specificity of the antibody. In Figure 9b, the purified recombinant protein has a molecular weight of approximately 23 kDa.

### 3.5. Colocalization of Mr-KIFC1 and Microtubules during Spermatogenesis of M. rosenbergii

**Figure 10:**
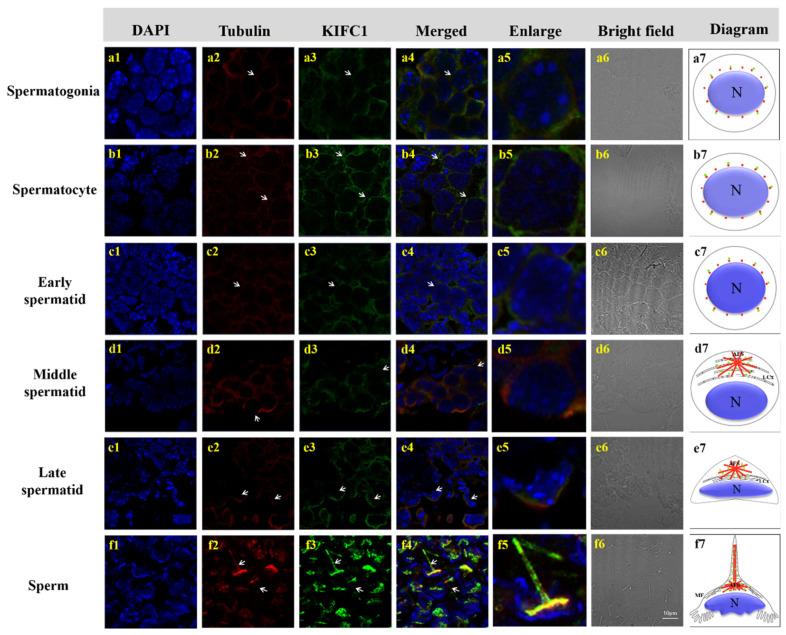
Immunofluorescence assay showing colocalization of Mr-KIFC1 and α-Tubulin in *M. rosenbergii* during spermatogenesis. (**a1**–**f1**) DAPI nuclear staining (blue); (**a2**–**f2**) α-Tubulin signal (red). (**a3**–**f3**) Mr-KIFC1 signal (green); (**a4**–**f4**) Merge of DAPI, α-Tubulin signal, and Mr-KIFC1 signal; (**a5**–**f5**) Higher magnification of (**a4**–**f4**,**a6**–**f6**) Phase contrast microscopy. (**a7**–**f7**) Spatial relative abundance of Mr-KIFC1 (green signal) and tubulin during spermatogenesis. (**a**) Spermatogonia: Mr-KIFC1 and α-Tubulin colocalize in the cytoplasm and near the nuclear membrane. (**b**) Spermatocytes: Nuclei larger than in the spermatogonia stage. The location of Mr-KIFC1 and α-Tubulin is the same as in the spermatogonia stage, but their signals are enhanced by comparison. (**c**) Early spermatid: Mr-KIFC1 and α-Tubulin colocalization in the cytoplasm surrounding the nucleus. (**d**) Middle spermatid: The nucleus moves to one end, α-Tubulin signals accumulate in the LCx in front of the cytoplasm, and Mr-KIFC1 signals colocalize with α-Tubulin in the area of the LCx. (**e**) Late spermatid: α-Tubulin is mainly localized in the AFS structure. Mr-KIFC1 located in the proacrosome associated with AFS. (**f**) Mature sperm: The AFS structure protrudes outwards to form a spinous process, and Mr-KIFC1 is distributed in the whole AFS structure, including the spinous process and colocalized with α-Tubulin. N: nucleus, LCx: lamellar complex, AFS: acroframosome, MF: microfilaments. Scale bar = 10 μm.

In *M. rosenbergii*, spermatogonia were ovoid in shape, with a large nucleus, nucleoplasm loosely distributed on the inner side of the nuclear membrane, cytoplasm rich in ribosomes and mitochondria, and α-Tubulin uniformly distributed throughout the cytoplasm around the nuclear membrane; Mr-KIFC1 colocalized with the α-Tubulin (Figure 10a1–a7). Spermatocytes were ovoid in shape. When the round nucleus was larger than in the spermatogonia period, chromatin was condensed, and α-Tubulin colocalized with Mr-KIFC1 in the cytoplasm (Figure 10b1–b7). Spermatids were divided into three stages according to the state of chromatin agglutination, namely, the early spermatid stage, middle spermatid stage, and late spermatid stage. Early spermatids are round in shape, and the nuclei are also round. Chromatin is uniformly distributed throughout the nucleus; the cytoplasm was rich in mitochondria, endoplasmic reticulum, and ribosomes, and Mr-KIFC1 colocalized with α-Tubulin in the cytoplasm around the nucleus (Figure 10c1–c7). In mid-stage spermatids, the cell gradually changed from a round to flat disk shape, the nucleus gradually expanded, the nuclear material continuously concentrated and moved forwards, a large amount of cytoplasm was distributed in front of the spermatocyte, and the endoplasmic reticulum, mitochondria, centrosomes, and Golgi vesicles distributed in the cytoplasm gathered together to form a temporary organelle LCx, at which time α-Tubulin was scattered in the LCx region. Mr-KIFC1 was localized in the whole area of the LCx (Figure 10d1–d7). In late-stage spermatids, chromatin was gradually concentrated, the nucleus at the posterior end was crescent-shaped, and the centrosome in the cytoplasm at the anterior end drove the formation of the AFS structure. α-Tubulin was mainly located in the AFS frame, and Mr-KIFC1 was located at the substrate structure associated with the AFS (Figure 10e1–e7). In mature sperm, the center of the AFS gradually elongated to form a spike-like structure, which was combined with its substrate, resembling an outwardly turned umbrella structure with a crescent-shaped nucleus at the posterior end. α-Tubulin was concentrated in the center of the acrosome, and Mr-KIFC1 was distributed throughout the acrosome, including the entire spike structure (Figure 10f1–f7).

### 3.6. Colocalization of Mr-Acrosin and Microtubules during Spermatogenesis of M. rosenbergii

**Figure 11:**
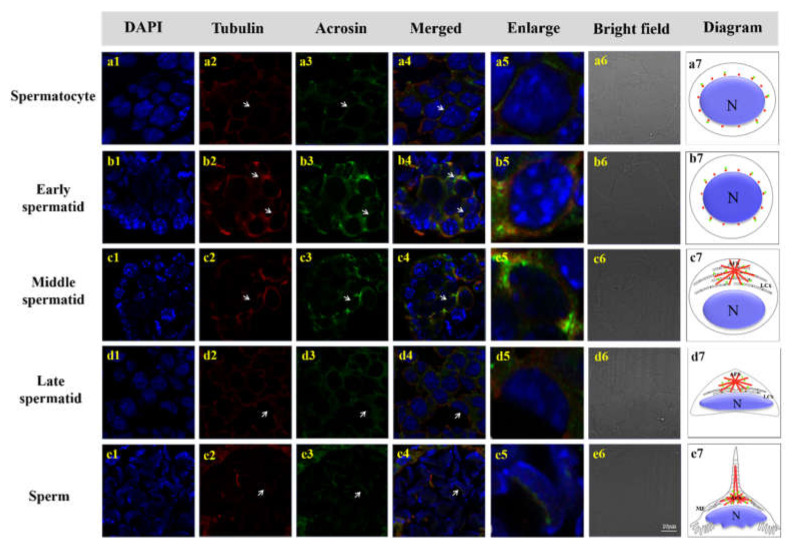
IF assay showing the colocalization of Mr-Acrosin and α-Tubulin during spermatogenesis. (**a**) Spermatocytes: Mr-Acrosin colocalizes with α-Tubulin in the cytoplasm surrounding the nucleus. (**b**) Early spermatid: Mr-Acrosin and α-Tubulin signals were increasingly colocalized in the cytoplasm and near the nuclear membrane. (**c**) Middle spermatid: Mr-Acrosin and α-Tubulin are mainly concentrated in LCx at the front of the cytoplasm, the signals of Mr-Acrosin are relatively more. (**d**) Late spermatid: α-Tubulin is distributed in the AFS, and Mr-Acrosin colocalizes with α-Tubulin. (**e**) Mature sperm: α-Tubulin signals arise in the AFS, but Mr-Acrosin signals appear only in the lower part of the AFS structure, not in the spinous process. Blue: DAPI, Green: Mr-Acrosin, Red: α-Tubulin. Scale bar = 10 μm.

Mr-Acrosin signals in spermatocytes and early spermatids were distributed in the cytoplasm around the round nucleus and colocalized with α-Tubulin signals (Figure 11a1–a7). In early spermatids, Acrosin signals were enhanced and more concentrated in the anterior part of the nucleus (Figure 11b1–b7). In mid-stage spermatids, Mr-Acrosin signaling was more pronounced at the acrosomal substrate (i.e., the LCx structure) (Figure 11c1–c7). In late spermatids, Acrosin signals clustered in the same AFS structure as α-Tubulin at the anterior end of the cytoplasm, and the nucleus was located at the posterior end in a crescent shape (Figure 11d1–d7). In mature sperm, Mr-Acrosin signals were concentrated at the substrate of the anterior convex end of the nucleus and the lower half of the AFS structure but did not appear in the spike structure (Figure 11e1–e7).

## 4. Discussion

### 4.1. Structural Features and mRNA Expression Characteristics of Mr-KIFC1 and Mr-Acrosin

Most kinesin superfamily proteins have three domains, namely, a motor domain, a coiled-coil domain, and a tail domain [26,27]. The motor domain consists of two globular structures, which are conserved in most species. It includes several ATP and microtubule binding sites, which are used to hydrolyze ATP for energy and bind to microtubules, respectively. The coiled-coil domain is composed of heavy chains connecting the motor domain and the tail domain [25,26]. However, the tail domains are divergent in different species, and they contain recognition sequences for regulatory kinases, other binding partner proteins, membrane organelles, and mRNAs [27,40]. KIFC1 is a member of the kinesin-14 family; it was first identified in the brain of mouse embryos and was later found to be abundant in mouse testes and also detectable in the liver, ovaries, and spleen [32,41]. In our present study, we obtained *Mr-kifc1* cDNA sequences from the testis of *M. rosenbergii*, compared them with the KIFC1 sequences from different species (Figure 2), and constructed an evolutionary tree (Figure 3), illustrating that KIFC1 has maintained a critical function throughout evolution. We found that Mr-KIFC1 has seven ATP binding sites and three microtubule binding sites in the motor domain (Figure 2), confirming that Mr-KIFC1 transports cellular products along microtubules. This is consistent with the evolutionary process, implying that Mr-KIFC1 has a fundamental role in microtubule association and cellular product transport. Meanwhile, we detected *Mr-kifc1* expression in all tissues, suggesting that Mr-KIFC1 plays an important role in *M. rosenbergii*. Our results showed that KIFC1 has relatively high expression in the testis compared with other tissues (Figure 7a,b). This finding is in alignment with our previous findings in other species [14,38,42], indicating that Mr-KIFC1 has a crucial role in the testis of *M. rosenbergii*.

Acrosin, a trypsin-like serine protease, was found in the spermatozoa of vertebrates and invertebrates [43,44]. It is located in the acrosome, a lysosome-like organelle, and covers the head of the sperm. Acrosin plays an important role in the acrosome reaction and the binding and penetration of the zona pellucida between sperm and ovum [19]. The Mr-Acrosin protein is composed of a long serine protease-trypsin domain and a clip domain (Figure 6). This is consistent with the characteristics of clip-serin proteases (clip-SPs). We detected *Mr-Acrosin* to be highly expressed only in the testis (Figure 7c,d), indicating its tissue expression specificity. *Mr-Acrosin* was stored in the AFS structure of *M. rosenbergii* and played an important role in later sperm–egg binding. At the same time, we found that *Mr-Acrosin* was also expressed in the heart (Figure 7c,d). We speculated that Acrosin has an important function in the heart, but this specific function remains to be studied.

### 4.2. Mr-KIFC1 Participates in Spermiogenesis and Is Closely Related to Nuclear Reshaping and Acrosome Formation

As a microtubule-dependent molecular motor protein, KIFC1 has been shown to assist in acrosome formation, nucleus reshaping, and flagellum formation during spermiogenesis [32]. In mammalian spermiogenesis, a skirt-like microtube structure named the manchette plays an important role. The manchette provides the track for KIFC1 movement, and KIFC1, manchette, and the nuclear pore protein Nup62 form a complex to assist in the transition from a round to an elliptical nucleus and participate in acrosome formation [45]. During the spermiogenesis of teleost *L. crocea*, KIFC1 interacted with manchette and importin β, participating in nucleus reshaping and acrosome formation. In addition, KIFC1 colocalized with mitochondria. KIFC1 is responsible for transporting mitochondria to the tail of the spermatid and is involved in flagellum formation [35]. In the spermiogenesis of amphibian *Cynops orientalis*, no manchette or manchette-like structures were observed. KIFC1 is involved in nucleus formation and the transport of material between nucleoplasms with the assistance of Nup62 and microtubules [46,47]. In the spermiogenesis of the crustacean *E. sinensis*, KIFC1 participates in sperm acrosome formation and nucleus maturation [48]. Previously, research reported that a unique microtubular structure called the AFS was formed during caridean shrimp spermatogenesis and that the AFS structure was similar to that of the manchette [15]. In the spermiogenesis of the caridean shrimp *M. nipponense* and *E. sinensis*, microtubules form a unique AFS structure, but the specific mechanism underlying its formation is still unknown. KIFC1 transports cargoes along the AFS and participates in acrosome formation and nucleus reshaping [8,14].

In our ISH results, we found that *Mr-kifc1* mRNA was strongly distributed in the cytoplasm around the nucleus in early spermatids. Afterwards, the spatial and temporal expression pattern of *Mr-kifc1* mRNA constantly changed as the nucleus was reshaped and acrosome formed (Figure 8). When the AFS structure was formed, *Mr-kifc1* mRNA was uniformly distributed in the AFS structure. This may provide more evidence for the role of *Mr-kifc1* mRNA in acrosome genesis and nucleus reshaping in *M. rosenbergii*, which is consistent with related studies in other species [32,35,38]. At the same time, this may indicate the locations of KIFC1 protein expression and the potential functions of KIFC1.

Based on the results of Mr-KIFC1 localization in each stage of spermatogenesis, we found that Mr-KIFC1 was localized in the cytoplasm around the nucleus in spermatogonia, spermatocytes, and early spermatids (Figure 10c3). The nucleus was deformed into an oval shape, and Mr-KIFC1 was localized in the LCx structure at the front of the nucleus in middle spermatids (Figure 10d3). These results may explain the function of Mr-KIFC1 as a motor protein involved in nuclear reshaping, which is consistent with previous research [32,36]. In late spermatids and mature sperm, Mr-KIFCI was localized in the gradually formed umbrella-shaped AFS structure, and the nucleus was deformed into a crescent shape and surrounded by the AFS structure (Figure 10e3,f3). This implied that the formation of the AFS structure depends on the temporary organelle LCx. At the same time, during the entire process of spermatogenesis in *M. rosenbergii*, Mr-KIFC1 localization was closely linked to nucleus reshaping (Figure 10a3–f3), which may explain the function of Mr-KIFC1 as a motor protein involved in sperm nucleus deformation. We also discovered that microtubules formed an umbrella-shaped AFS structure, and Mr-KIFC1 signals overlapped with tubulin, which may suggest that AFS act as scaffolds for Mr-KIFC1 to transport cargoes that are later organized into functional acrosomes. Therefore, we speculated that Mr-KIFC1 transports different cargos along the AFS structure to participate in nuclear reshaping and acrosome formation during the spermatogenesis of *M. rosenbergii*.

### 4.3. Mr-KIFC1 Is Involved in Mr-Acrosin Transport in Spermatogenesis

How does KIFC1 participate in acrosomal formation? In this study, we found that the spatial and temporal distributions of Mr-KIFC1 and Mr-Acrosin were extremely similar (Figure 11). KIFC1 could transport membrane organelles [49], protein complexes [50], vesicles [51], RNA [40], and dsDNA [52] to specific sites for their functions. Acrosin is a serine protease present in the acrosome that has been most extensively studied in mammals [44]. Acrosin is usually present in the acrosome in the form of the inactive precursor proacrosin [53], which is activated during the sperm–egg union process [18]. The acrosome releases Acrosin to hydrolyze the zona pellucida and yolk membrane around the egg, allowing the sperm–egg union to complete the fertilization process to form a fertilized egg [54,55]. Acrosin promotes the release of myostatin in the reproductive system, enhances sperm motility, and promotes sperm movement [19,56]. Although crustacean sperm acrosome morphology differs significantly from that of mammals, acrosome origin is evolutionarily conserved [57]. It has been shown that Acrosin can act as a marker of the acrosome during spermatogenesis in *E. sinensis* and function as a hydrolytic zona pellucida during fertilization [58]. During spermatogenesis in most vertebrates and the crustacean *E. sinensis* [43,44,53], it has been demonstrated that Acrosin signals first appeared in haploid spermatocytes and gradually increased during differentiation from haploid spermatocytes to sperm.

Our immunofluorescence results showed that the localization of Mr-Acrosin signals first appeared in secondary spermatocytes (Figure 11a3), suggesting that Acrosin is not associated with the first meiotic division, consistent with previous studies [43,44,53]. Mr-Acrosin may play a role in the second meiotic division and spermiogenesis. From secondary spermatocytes to early spermatids, Mr-Acrosin was clustered around the nucleus, colocalized with Mr-KIFC1, and the signal was gradually enhanced, after which the position of acrosome formation was gradually ectopic (Figure 10b3,c3 and Figure 11a3,b3), illustrating that Mr-Acrosin may play an important role in the second meiotic division or in preparation for subsequent spermiogenesis. Since Mr-Acrosin is colocalized with KIFC1, Mr-Acrosin may be transported by Mr-KIFC1. In middle spermatids, Mr-Acrosin signals were clustered in the LCx structure, similar to the Mr-KIFC1 signals (Figure 10d3 and Figure 11c3). The LCx is related to acrosome formation [14], so we hypothesized that Mr-Acrosin may be involved in acrosome formation through the LCx. In late spermatids and mature sperm, Mr-Acrosin signals were observed to colocalize with Mr-KIFC1 signals in the acrosomes of the AFS structure (Figure 10e3,f3 and Figure 11d3,e3). During spermatogenesis, KIFC1 could transport different cargoes to the designated site [25,26,27,59]. The above results indicate that Mr-Acrosin and Mr-KIFC1 are both involved in spermatogenesis and that Mr-Acrosin is transported by Mr-KIFC1, which leads us to speculate that Mr-KIFC1 may transport Mr-Acrosin along the AFS structure to the acrosome to exert protease-like effects and participate in the sperm–egg union in *M. rosenbergii*.

## 5. Conclusions

In our present study, we obtained full-length *Mr-kifc1* and *Mr-Acrosin* from the testes of *M. rosenbergii*. The protein structure and phylogenetic evolutionary tree showed that Mr-KIFC1 is strongly evolutionarily conserved. Abundant *Mr-kifc1* and *Mr-Acrosin* mRNA transcripts were detected in the testis of *M. rosenbergii*. The location of *Mr-kifc1* mRNA transcripts was consistent with that of Mr-KIFC1. Mr-KIFC1, microtubules, and the AFS structure play important roles in the spermatogenesis of *M. rosenbergii*. Mr-KIFC1 participated in nuclear reshaping and acrosome formation. The colocalization of Mr-KIFC1, α-Tubulin, and Mr-Acrosin indicated that Mr-KIFC1 transports Mr-Acrosin along the AFS structure, allowing Mr-Acrosin to be transported to a designated location in the acrosome, facilitating participation in the lysis of the oocyte membrane. However, more studies on the specific mechanism of Acrosin transport by KIFC1 need to be performed.

This study provides a model for studying the mechanism of spermatogenesis in crustaceans and advances our knowledge of the reproductive biology of crustaceans. This study is the first to propose the function of KIFC1 to transport acrosomal enzymes along the AFS structure during crustacean spermatogenesis. This provides a reference for studying the kinesin transport of different cargoes.

## Figures and Tables

**Table 1:** The primer sequence used in this research.

Primer Name	Sequence (5′ to 3′)	Purpose
KIFC1-F	GAGCAGCTTGGGAYYTNAARGG	PCR (kifc1 cloning)
KIFC1-R	CCWGTYTGTCCRTANGCRAA	PCR (kifc1 cloning)
5′ KIFC1-R	TCCAGGTTGGATTTGGAGAACGTGAGAC	5′ RACE (kifc1 cloning)
3′ KIFC1-F	CAAGGGGAGATGGTGCGGAGA	3′ RACE (kifc1 cloning)
KIFC1-BDLF KIFC1-BDLR BDKIFC1-F1 BDKIFC1 R1	AACGCCAGGTCAACTCCC GAAATGATGGCACAAATAGAAG CGCGGATCCATGCCAAAGCTCCCTACTT CCGGAATTC CTGCTGATTCCTCTCCACC	qPCR qPCR Antibody Antibody
ACR-F1	GGGGAGCACAACTTGATCACAGAGAGAGA	PCR (acr cloning)
ACR-F2	TGCTCATTGCTTCAAGAAACAACTGGGA	PCR (acr cloning)
ACR-R1	GGGGAACCACAGCCAGGGAT	PCR (acr cloning)
ACR-R2 ACR-R3	TCCAATCCACGAGACGCCAT GCCCTGAAAGGAAGTTACCCA	PCR (acr cloning) PCR (acr cloning)
5′ACR-R1 3′ACR-F1 UPM-long UPM-short NUP 3′Outer 3′Inner BD-Acr-F1 BD-Acr-R1Acrosin-BDLFAcrosin-BDLR β-actin-F β-actin-R	ACCCTTGAGTTTGCCAGAAT GCAGGAGGCGAGGGTAAAGA CTAATACGACTCACTATAGGGCAAGCAGTGGTATCAACGCAGAGT CTAATACGACTCACTATAGGGC AAGCAGTGGTATCAACGCAGAGT TATTGGGCTGATTCTTGATGACA CGCGGATCCTCCACTAGTGATTTCACTATAGG CCGGAATTCAGCTTCTGGTTCTGTGGAG CCGCTCGAGCTTGTTTGGGTAACTTCCT GAGCTTCTGGTTCTGTGGAG CCTTGTTTGGGTAACTTCCT CAGGAATCGCTGACAGAATG GAAGGTAGAAAGAGAAGCCAAGA	5′ RACE (acr cloning) 3′ RACE (acr cloning) 5′ RACE (cloning) 5′ RACE (cloning) 5′ RACE (cloning) 3′ RACE (cloning) 3′ RACE (cloning) Antibody Antibody qPCR qPCR Positive control of qPCR Positive control of qPCR

## Data Availability

*Mr-kifc1* nucleic acid sequence has been uploaded to NCBI (GenBank: JN627516.1). *Mr-Acrosin* nucleic acid sequence has been uploaded to NCBI (GenBank: OL840042).
